# Development of a selected reaction monitoring mass spectrometry-based assay to detect asparaginyl endopeptidase activity in biological fluids

**DOI:** 10.18632/oncotarget.12224

**Published:** 2016-09-23

**Authors:** Anindita Dutta, David N. Potier, Michael J. Walker, Oliver J. Gray, Catriona Parker, Mark Holland, Andrew J.K. Williamson, Andrew Pierce, Richard D. Unwin, Shekhar Krishnan, Vaskar Saha, Anthony D. Whetton

**Affiliations:** ^1^ Stem Cell and Leukaemia Proteomics Laboratory, Faculty of Biology, Medicine and Health, University of Manchester, Manchester, UK; ^2^ Tata Translational Cancer Research Centre, Kolkata, India; ^3^ Stoller Biomarker Discovery Centre, Faculty of Biology, Medicine and Health, University of Manchester, Manchester, UK; ^4^ Children's Cancer Group, Faculty of Biology, Medicine and Health, University of Manchester, Manchester, UK; ^5^ Current address: Centre for Advanced Discovery and Experimental Therapeutics, Central Manchester University Hospitals NHS Foundation Trust and Institute of Human Development, University of Manchester, Manchester, UK

**Keywords:** legumain, AEP, biomarker, SRM, protease

## Abstract

Cancer Biomarkers have the capability to improve patient outcomes. They have potential applications in diagnosis, prognosis, monitoring of disease progression and measuring response to treatment. This type of information is particularly useful in the individualisation of treatment regimens. Biomarkers may take many forms but considerable effort has been made to identify and quantify proteins in biological fluids. However, a major challenge in measuring protein in biological fluids, such as plasma, is the sensitivity of the assay and the complex matrix of proteins present. Furthermore, determining the effect of proteases in disease requires measurement of their activity in biological fluids as quantification of the protein itself may not provide sufficient information. To date little progress has been made towards monitoring activity of proteases in plasma. The protease asparaginyl endopeptidase has been implicated in diseases such as breast cancer, leukaemia and dementia. Here we describe a new approach to sensitively and in a targeted fashion quantify asparaginyl endopeptidase activity in plasma using a synthetic substrate peptide protected from nonspecific hydrolysis using D-amino acids within the structure. Our selected reaction monitoring approach enabled asparaginyl endopeptidase activity to be measured in human plasma with both a high dynamic range and sensitivity. This manuscript describes a paradigm for future development of assays to measure protease activities in biological fluids as biomarkers of disease.

## INTRODUCTION

Detailed investigations of the altered biological processes in diseases have allowed the identification of specific genes/proteins involved in the pathological process. Potentially these molecules can be used as biomarkers. Ideal clinically relevant biomarkers should be easy to measure, show wide dynamic range and be measured in peripheral blood samples as these have ease of collection and potential serial sampling in their favour [1, 2]. For example, levels of prostate-specific antigen and cancer-antigen 125 are used to diagnose and monitor prostate and ovarian cancers respectively [3, 4]. However, measuring proteins in plasma is challenging, since the dynamic range of proteins present in plasma spans over ten orders of magnitude [5]. This large dynamic range makes biomarker discovery studies difficult, as high abundant proteins mask the lower abundant proteins. Moreover, when the marker in question is an enzyme what may be more relevant is a measure of its activity. An example of this is the cysteine lysosomal protease asparagine endopeptidase (AEP) which has high specificity for cleavage at the carboxyl terminal of asparagine residues [6–8]. AEP is synthesized as a zymogen and converted to an active form in the extracellular matrix by the act of autocatalysis under acidic conditions [9]. Maximal activity of AEP is observed at an acidic pH of 5.8 while its activity is lost below pH 4.5 and above pH 7 [10]. In cases of breast, prostate and colon cancers aberrant overexpression of AEP correlates with invasion, dissemination and poor outcome [11–13]. We have shown that overexpression of AEP is seen in high risk cytogenetic subtypes of childhood acute lymphoblastic leukaemia (ALL) [14]; correlates with CNS infiltration in ALL [15] and that the enzyme cleaves and degrades a key anticancer drug used in the treatment of ALL [9]. AEP expression is reported as an independent predictor of poorer overall survival in Asian patients with breast cancer [16] and colon cancer [11]. Serum AEP levels were significantly higher in patients with breast cancer compared to the healthy normal and was correlated with poor survival [12]. Higher levels of serum AEP are also associated with tumor invasion and migration *in vivo* [17]. In patients with Alzheimer's disease, elevated levels of neuronal AEP activation has been reported to contribute to synaptic loss by mediating Tau hyper-phosphorylation and consequently microtubular disruption [18]. Monitoring protein levels therefore may not be sufficient to characterise the disease process or the molecular pathology associated with dysregulation of this enzyme. Mutations of AEP at its active site, such as those occurring at H150 (H150A) and C191 (C191S) can virtually eliminate all AEP activity [19] and this is not detectable by conventional ELISA assays. Current AEP activity assays commonly use fluorescence based quantification [10, 15], involving incubation of synthetic AEP-specific substrates in samples for a fixed period of time. Such fluorescence assay based approaches have limited sensitivity and dynamic range and because of the natural fluorescence of high abundance proteins such as albumin [20] have limited applicability in plasma samples. Targeted quantitative Mass Spectrometry (MS) using selected reaction monitoring (SRM) [21], is highly sensitive and selective and can be used for the quantification of specific small molecules, such as drug metabolites [22]. Here we describe a novel SRM-based method to monitor AEP activity in plasma which can be applied to biofluids and shows high dynamic range, reproducibility and sensitivity.

## RESULTS AND DISCUSSION

### Assay design

**Figure 1:**
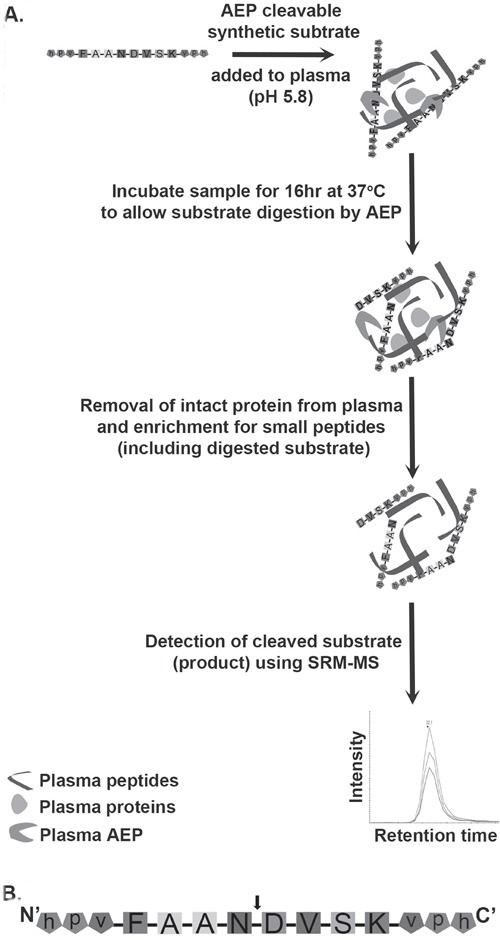
Schematic diagram of the workflow involved in assaying AEP activity in plasma samples **A.** The workflow to measure AEP activity in plasma. AEP cleavable synthetic substrate was added to plasma at an acidic pH (pH 5.8) followed by an incubation at 37°C for 16 hr. This incubation allows endogenous plasma AEP to interact with synthetic substrate and cleaves substrate into specific products. Removal of intact proteins from plasma using acetonitrile (ACN) precipitation gives enrichment of peptides including products formed by digestion of synthetic substrate by AEP. The cleaved form of substrate is detected by mass spectrometer following SRM based approach. **B.** AEP cleavable synthetic substrate peptide (FAANDVSK) was designed and synthesized with a cleavage site for AEP at the C-terminal of asparagine (N) residue. The arrow head represents the AEP cleavage site. Both the N and C terminals of the substrate were protected by using a D-amino acid capping (represented by lower case).

There is a clinical need for assays to measure enzyme activity in biological fluids. We therefore set out to design a reproducible and sensitive assay with high dynamic range to monitor AEP activity as a paradigm study. The SRM-based method is outlined in Figure 1A. The workflow plan was based on addition of a specific AEP cleavable synthetic peptide to plasma at a pH of 5.8 (optimum for AEP activity) to allow endogenous AEP in the plasma to act on the synthetic substrate. After peptide enrichment using a method we have previously developed [23], samples were to be analysed by SRM-MS to quantify the amount of cleaved substrate. Plainly a critical aspect of this SRM-MS based assay relies on designing the appropriate AEP targeted synthetic peptide using available data.

### Designing AEP cleavable synthetic substrate

Several important criteria were taken into account while designing the synthetic substrate. Firstly, the product formed by AEP activity had to be non-endogenous in humans. The presence of any endogenous cleaved product would lead to errors in quantifying the actual level of AEP activity as the amount of naturally occurring peptide in plasma from different individuals will vary. In order to design the synthetic substrate the known cleavage sites for all substrates of AEP recorded in the literature were examined using the MEROPS database [8]. Based on this data, it was evident that an asparagine residue is essential at the P1 position. In addition, AEP appears to have a preference for peptides containing alanine at the P2 and P3 positions, as well as a phenylalanine residue at the P4 position. From this analysis the peptide sequence FAANDVSK (Figure 1B) was chosen as an AEP specific target peptide. AEP cleavage was anticipated to take place between the N and D amino acid residues which results in the products FAAN and DVSK.

Next, steps were taken to ensure non-specific proteolytic digestion of the peptide was avoided. Villanueva *et. al.* demonstrated that proteolytic digestion is inhibited at the non-naturally occurring D-isoform of an amino acid [24]. By capping both ends of the target peptide with a stretch of three D-amino acids, the effect of exopeptidases on the target synthetic peptide would be minimized (represented in lower case, Figure 1B). In the first instance the peptide was capped at each end with amino acid sequences hpv, hav or hph. Histidine residues were selected as they can sequester positive charge in electrospray ionisation, which should aid in detecting the cleaved forms of the target peptide in the SRM analyses. *In silico* analysis of the amino acid sequences of the peptides, hphFAAN, hpvFAAN, havFAAN, DVSKhph, DVSKvph and DVSKvah indicated that none of these product peptides, generated upon cleavage of synthetic substrate, are found endogenously in humans.

### Synthetic substrate peptide and its products are detectable through MS

**Figure 2:**
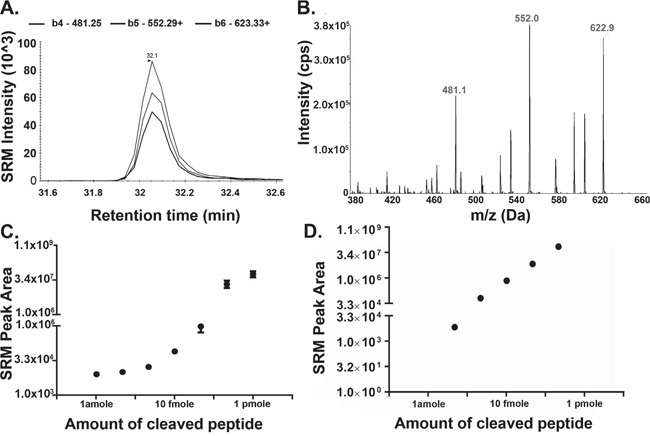
Detection of Cleaved Substrate Peptide by Mass Spectrometry **A-B.** 10 ng of recombinant AEP (rAEP) was incubated with 1 μg (0.65 mM) synthetic substrate peptide (hpvFAANDVSKvph) overnight at 37°C. Samples were dried down by vacuum centrifuge and resuspended in LB. 5 pmole of the substrate was loaded on to the liquid chromatography column and cleaved substrate was analysed by SRM MS (A) followed by an DDA workflow (B). (A) Three SRM transitions (b4-481.25^+^, b5-552.29^+^, b6-623.33^+^) were used to detect the cleaved substrate, sequenced as hpvFAAN (378.2^2+^ precursor ion). The target peptide eluted at 32 min during the chromatographic gradient. (B) Product ion scans of precursor 378.2^2+^ as analysed by DDA. **C.** Synthetic peptide sequenced as cleaved form of the substrate (hpvFAAN) was dissolved into HPLC grade water and seven different concentrations from 1 amole to 1 pmole were made in loading buffer (LB), containing 20 mM citric acid, 0.1% FA, 0.1% ACN. **D.** 10 amole, 100 amole, 1 fmole, 10 fmole, or 100 fmole of the substrate (hpvFAAN) peptide was spiked into normal plasma. Samples were precipitated using 2 volumes of ACN and dried down using vacuum centrifugation. Samples were resuspended in LB and analyzed by SRM MS. Error bars shown are +/−SEM (n = 3).

The synthetic substrates, designed *in silico* (hphFAANDVSKhph, havFAANDVSKvah, hpvFAANDVSKvph), were tested for their ability to be selectively hydrolysed into products with appropriate chromatographic and mass spectral properties. Incubation of the synthetic substrates along with recombinant AEP (rAEP), allowed testing for cleavage and product analysis. Samples were assessed using SRM followed by data-dependent acquisition (DDA) workflows. Theoretical transitions for each intact substrate and also for their corresponding products were generated. The peptide hphFAANDVSKhph was found to be highly hydrophilic with poor retention on reverse phase liquid chromatography columns and thus a very early elution time during the gradient (data not shown), and was not pursued any further in the study. Cleavage of the synthetic substrate, havFAANDVSKvah produced havFAAN and DVSKvah fragments. The havFAAN fragment eluted at 32 min during the liquid chromatography process; however, the intensity of the SRM transitions was low and DDA did not confirm the transitions observed by the SRM-MS method (data not shown). AEP-mediated cleavage of hpvFAANDVSKvph generated hpvFAAN and DVSKvph as products. Three transitions for the product “hpvFAAN” doubly charged precursor ion (m/z 378.2^2+^) were detected at 32 min during the chromatographic gradient using the MS method (Figure 2A). The three transitions observed in SRM were confirmed by DDA workflow with enhanced product ion scanning for fragmentation of the hpvFAAN producing singly charged ions at m/z 481.1, 552.0 and 622.9 at 32 min retention time (Figure 2B). Peptides naturally present in plasma with the identical SRM transitions of the synthetic intact or cleaved substrate peptide, could potentially interfere with this assay. To evaluate this, three SRM transitions for the cleaved substrate (hpvFAAN) were tested on control plasma collected from healthy individuals. This showed minimal responses in a standard LC-MS run indicating no similar endogenous peptides were present in plasma at the retention times for the cleaved substrate (Supplementary Figure S1). The product hpvFAAN, generated by the cleavage of the synthetic substrate (hpvFAANDVSKvph), was therefore used for the further development of the AEP activity assay.

### Cleaved form of the synthetic substrate is stable in plasma

For an assay to measure enzyme activity it is important that the product is stable for the duration of the assay and is present at a level that can be detected. The limit of detection (LOD) and stability of the product hpvFAAN were analysed by serial dilution down to 1 amole in the presence or absence of plasma using SRM MS analysis. This allowed the LOD to be calculated as 2 amole with a linear response up to 1 pmole without a plasma background (Figure 2C). The effect of a plasma matrix increased the LOD to 10 amole (Figure 2D) but the dose response was still linear.

### Recombinant AEP activity in human plasma

**Figure 3:**
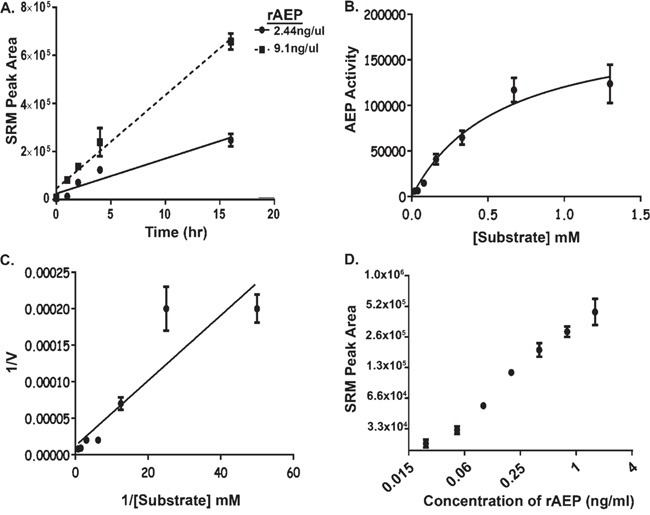
Development of Assay Conditions to Monitor AEP Activity in Plasma **A.** The level of hydrolysis of AEP substrate with respect to time. 2.44 ng/μl and 9.1 ng/μl of rAEP were spiked into a background of pH modified plasma (pH 5.8) and assayed by SRM-MS for the product peptide. Error bars shown are +/−SD (n = 3). **B-C.** Rate of AEP activity assayed with different concentrations of substrate (hpvFAANDVSKvph) ranging from 0.02 mM to 1.3 mM. Area under the curve, drawn from SRM-MS of the product peptide, was used to obtain the rate of reaction for AEP activity as shown in Michaelis-Menten Curve (B). Lineweaver-Burk plot was generated to calculate the Km value (0.4 mM) for the synthetic substrate (C). “V” represents amount of product formed per hour. Error bars shown are +/−SD (n = 3). **D.** The linearity and limit-of-detection of the AEP assay was investigated by measuring the amount of hydrolysis by 2.0 pg to 0.25 ng of rAEP spiked into 25 μl of plasma and product measured by SRM-MS of Error bars shown are +/−SEM (n = 3). The assay was repeated three times and a technical variance of 3-28% was calculated.

In order to determine the optimum conditions to assay AEP activity in plasma, recombinant human AEP (2.44 ng/μl or 9.1 ng/μl) was spiked into normal plasma collected from healthy individuals and incubated with 0.67 mM of the synthetic substrate (hpvFAANDVSKvph) for 0 to 24 hr at 37°C. Product was detectable after 1 hr of incubation with a linear response observed up to 16 hr of reaction for both 2.44 ng/μl (r^2^=0.92) and 9.1 ng/μl (r^2^=0.988) of rAEP (Figure 3A). The level of product was higher after 16 hours for both enzyme amounts.

To optimise substrate concentration in our assay, 10 ng of rAEP was incubated with 0.02 mM to 1.3 mM of synthetic substrate for 1 hr, 4 hr (Supplementary Figure S2) or 16 hr (Figure 3B and 3C) at 37°C. The rate of reaction was determined from the amount of product formed per hour and used for Michaelis-Menten enzyme kinetic analysis (Figure 3B). A Lineweaver-Burk plot was generated to determine the Km value for the substrate (Figure 3C). The calculated Km values were 0.3 mM, 0.33 mM and 0.4 mM using the data from 1 hour; 4 hours and 16 hours respectively. As the 16-hour incubation had a linear response and generated the highest quantity of product, this incubation period was used in subsequent studies along with a substrate concentration of 4 mM, ten times in excess of the Km value to ensure the enzyme was assayed at V_max_. When tested in plasma samples spiked with rAEP the assay detected AEP activity from 0.02 ng/ml rAEP with a linear response between 0.04 ng/ml to 2.5 ng/ml (Figure 3D).

One of the greatest challenges in analyzing the plasma protein is the wide range of concentration of the proteins. A previous study reported more than 10 logs of molar abundance of specific proteins in plasma [25]. In our assay, we have overcome the dynamic range problem of the plasma proteome to detect AEP sensitively.

### Endogenous AEP activity in human plasma samples from patients with cancer

**Figure 4:**
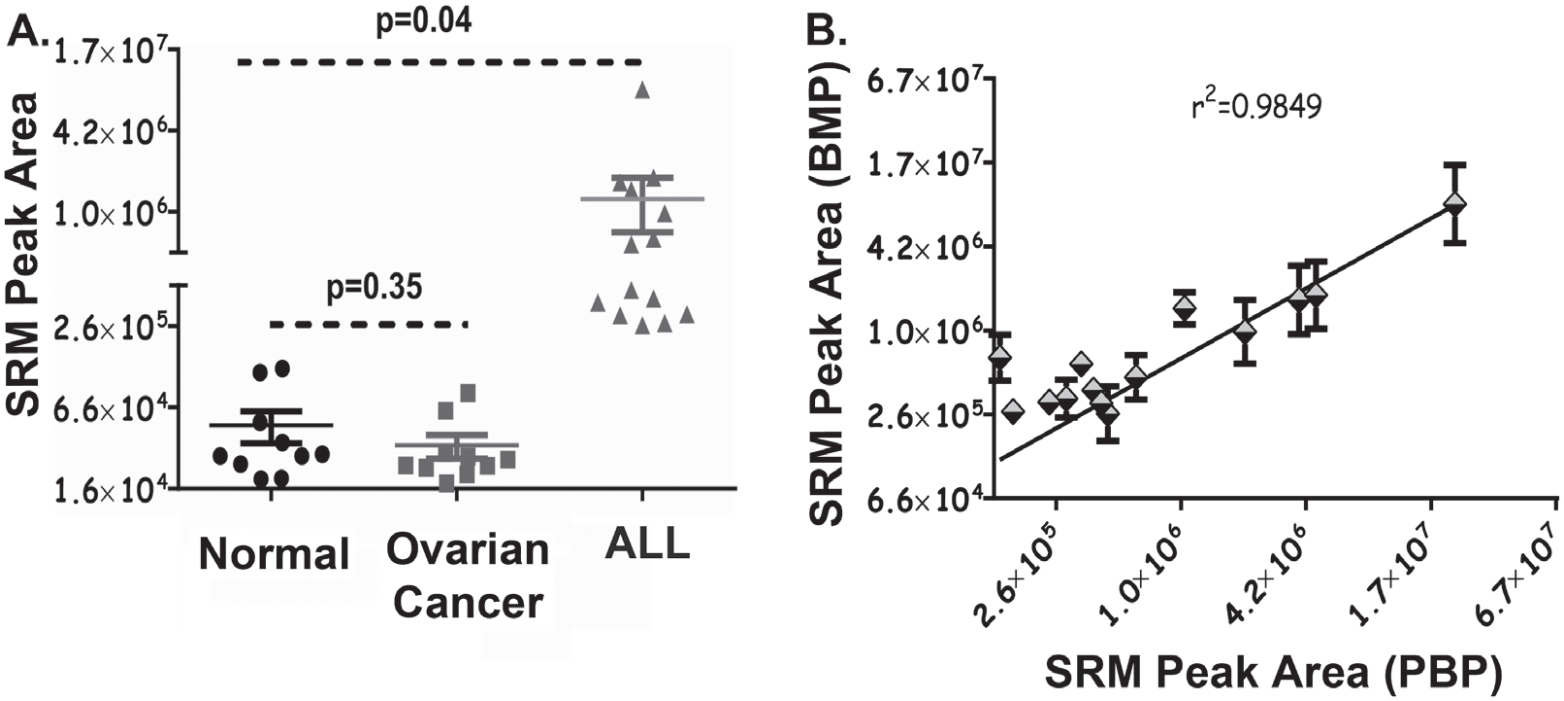
AEP activity in Human Plasma Collected from Normal and Diseased Individuals **A.** 25 μl of peripheral blood plasma samples (pH 5.8) from 10 healthy normal and 10 patients with ovarian cancer plus bone marrow plasma from 14 patients with ALL were measured for AEP activity using the assay developed. Samples were incubated with 4 mM AEP synthetic substrate at 37°C for 16 hr. Reactions were stopped by adding 2 volume of ACN. Enriched pool of small peptides was dried and resuspended in LB. Samples were analysed by the SRM-MS workflow. SRM peak area represents AEP activity in the plasma. Error bars shown are +/−SD. **B.** AEP activity was measured in 14 matched peripheral (PBP) and bone marrow (BMP) plasma samples collected from childhood patients diagnosed with ALL. 25 μl of plasma samples were used and pH was adjusted to 5.8. Samples were incubated with 4mM AEP synthetic substrate at 37°C for 16 hr, followed by protein precipitation using 2 volume of ACN. Enriched pool of small peptides was dried and reconstituted in LB. Samples were analysed by the SRM-MS workflow. SRM peak area for peripheral and bone marrow plasma is representative of AEP activity in respective samples. Error bars shown are +/−SD.

To test the utility of the AEP assay the enzymatic activity of AEP was measured in plasma samples from healthy volunteers and those with ovarian cancer or ALL by the SRM workflow and AEP protein levels measured using a commercially available ELISA kit. AEP levels in serum, measured using ELISA, have been previously reported to be in the order of approximately 10 ng/ml, with a slight increase shown in patients with benign fibroadenoma, and a significant increase to approximately 300 ng/ml in malignant carcinoma patients [12]. Here the ELISA assay detected quantities of AEP at 0.14 ng/ml to 6.8 ng/ml and 0.07 ng/ml to 2.7 ng/ml in plasma samples obtained from normal volunteers and ovarian cancer patients respectively (Supplementary Table S1). AEP levels varied from 0.13 ng/ml to 7.2 ng/ml in plasma from patients with ALL as detected by ELISA (Supplementary Table S2). However, it is biologically more significant to assay the activity of an enzyme (AEP in our study) rather than its amount in human plasma. AEP activity was measured in peripheral blood plasma from normal volunteers or those with ovarian cancer and in bone marrow plasma from patients with ALL. The SRM-MS based assay detected AEP activity in all the samples with differential levels of AEP activity being observed in normal and diseased plasma (Figure 4A). Though the levels of total plasma AEP were comparable, the SRM based assay significantly distinguished elevated AEP activity in the plasma from children with ALL when compared to AEP activity from controls.

Having demonstrated the utility of our assay we next determined whether we could use peripheral blood plasma to measure AEP activity in ALL. Whilst ALL is primarily a disease of the bone marrow, measurement of AEP activity in peripheral blood would constitute a much less invasive procedure. We measured AEP activity in 14 matched bone marrow and peripheral blood plasma collected from children with ALL at diagnosis. AEP activity obtained in bone marrow plasma positively correlated with AEP activity in peripheral plasma (r^2^= 0.9849). This suggests that AEP activity measured in the peripheral blood (a less invasive procedure) by SRM-MS is indicative of the enzyme activity at the site of tumour origin in childhood ALL (Figure 4B). Our novel approach, assessed by a number of parameters including measurement of limit of detection, linear range and variance for AEP activity, demonstrate effective assay improvement by using an SRM-MS based approach. Until now, no assays have been available with this dynamic range and sensitivity, limiting the use of AEP as a biomarker. This novel SRM-MS assay offers a minimally invasive method to monitor the biological activity of AEP in dementia and oncology research. AEP has been implicated in breast cancer pathogenesis and has been reported to have both prognostic and therapeutic value [12, 26]. A prodrug strategy using AEP cleavage to activate the compound has been developed which is effective in murine models [17, 27]. Our assay would provide a relatively cheap and minimally invasive method to select the patients that would benefit from this type of treatment in any clinical trial.

Plasma is the primary clinical specimen and it contains secreted tissue proteins thus it is of immense value as a source of biomarkers. Measuring protease activity in plasma offers new avenues for biomarker development. Therefore we designed an assay that uses synthetic peptide with D-amino acid isoforms to develop a paradigm approach to plasma enzymatic protein assay. In addition to measuring AEP activity the novel approach designed here can be utilised as template for further methods to detect the activity of other proteases involved in disease pathology. Indeed the ability to multiplex SRM assays offers the potential to monitor several clinically relevant proteases in one highly sensitive assay using several distinct peptide substrates. Incubation of plasma samples with a range of different synthetic substrate peptides designed for respective proteases will allow monitoring the activity of different proteases in a single assay using SRM-MS.

## MATERIALS AND METHODS

### Chemicals

Reagents were supplied as follows: HPLC grade water and 0.1% (w/v) formic acid with water, dithiothreitol (DTT) and 3-[(3-cholamidopropyl) dimethylammonio]-1-propanesulfonate (CHAPS) were from Sigma-Aldrich, St. Louis, MO; HPLC grade Acetonitrile (ACN) and hydrochloric acid (HCl) (VWR, Leicestershire, UK); Recombinant human AEP (rAEP), expressed in NS0 derived murine myeloma cell line, containing amino acids Ile18 to Tyr433 with an N-terminal 7 His tag, (R & D Systems, Abingdon, UK); AEP specific target peptides “hphFAANDVSKhph” (New England Peptides, Gardner, MA), “hpvFAANDVSKvph” and havFAANDVSKvah” including cleaved substrate “hpvFAAN” (JPT Innovative Peptide Solutions, Berlin, Germany); AnalaR grade Citric acid, di-sodium hydrogen phosphate (Na_2_HPO_4_) and Ethylenediaminetetraacetic acid (EDTA) (DBH, UK) and Total Legumain DuoSet ELISA kit (R&D Systems).

### Human plasma

Excess bone marrow aspirate and peripheral blood samples were obtained at diagnosis and at specific time points, from children aged 1 to 18 years, with pre-B-cell ALL enrolled to the UKALL 2003 trial (ISRCTN 07355119) between December 2007 and January 2011. Consent was obtained for the storage and use of excess material for ethically approved research. (Scotland A REC [ref 02/10/052]). Biomarker analyses of these samples were approved by North West 8 REC - GM East [ref 07/Q1402/56] research ethics committees. Plasma from ten healthy volunteers and patients with ovarian cancer were obtained from Seralab (Haywards Heath, UK).

### Enzyme activation

Recombinant human AEP (R & D Systems, Abingdon, UK) was activated as previously reported [28]. Briefly, 10 μg of enzyme was dissolved in assay buffer (50 mM sodium citrate solution, 5 mM dithiothreitol, pH 4.5), and incubated at 37°C for two hours.

### Liquid chromatography and mass spectrometry analysis

All samples were analysed on a 6500 Q-TRAP (AB Sciex, Warrington, UK) coupled to a nano Acquity uPLC (Waters Inc, MA, US). Peptides were resolved by a 75 mm x 250 mm Acquity uPLC BEH C18 column with 130 Å pore size after trapping with a 180 mm x 20 mm Symmetry C18 column with 5 mm diameter (Waters Inc, MA, US). Buffer A consisted of 99.9% water and 0.1% (v/v) formic acid. Buffer B consisted of 99.9% (v/v) ACN and 0.1% (v/v) formic acid. 20 mM citric acid with 0.1% ACN and 0.1%FA was used as loading buffer. Peptides were loaded at 2 μl/min for 10 min prior to being eluted over a 40 minute gradient at 0.3 μl/min (Table 1).

**Table 1:** The liquid chromatography gradient conditions to detect the AEP-cleaved synthetic peptide through MS

Time (min)	Buffer A (%)	Buffer B (%)
0	99.9	0.1
10	99.9	0.1
40	60.0	40.0
42	15.0	85.0
45	15.0	85.0
50	99.9	0.1

10 μl of samples were injected to analyse through LC-MS were loaded on the trap column for 10 min with a flow rate of 2 μl/min. Following 40 min during the gradient percentage of buffer B was increased to 40%. Peptides are expected to elute off during this time. Buffer B was further increased to 85% at 42 min along the gradient and continued till 45 min. The gradient was set back to initial status at 50 min.

In all MS analysis, the de-clustering potential was set at 90, the ion source voltage was 2500 V, the curtain gas was 20, the interface heater temperature was 150°C, and Quadrupole 1 and Quadrupole 3 were set to low and unit resolutions respectively. The dwell time for each transition was 50 ms. Data were integrated using the summation algorithm for all three transitions in Skyline-daily (beta) software (MacCoss Lab Software, WA, US).

### Cleaved substrate SRM generation

SRM transitions were generated for the N-terminal form of the cleaved AEP target synthetic, sequenced as hpvFAAN, havFAAN or hphFAAN using Skyline (Table 2). Transitions were confirmed using data dependant analysis (DDA) of 5 pmol of the peptide targets. The amount of product formed by the cleavage of the synthetic substrate was considered to be proportionate to AEP activity if the assay showed consistent rate of production over the time course and the substrate was present at a concentration above enzymatic V_max_.

**Table 2:** SRM transitions used to detect hphFAAN product

Peptide	Precursor ion	Product ion	Amino acid
hpvFAAN (2+)	378.195	481.255	F[b4]
	378.195	552.292	A[b5]
	378.195	623.330	A[b6]

SRM transitions were generated *in silico* using “Skyline-daily (beta)” software from MacCossLab Software, WA, US.

### Target peptide digestion and detection

The AEP specific target synthetic peptides (1 μg) were incubated with 20 ng of activated rAEP in assay buffer at 37°C for time stated. The samples were dried to completion and re-suspended in loading buffer (20 mM citric acid, 0.1% ACN, 0.1% FA (v/v)).

### Background SRM response in plasma

Plasma (25 μl) was diluted fivefold in assay buffer (containing 39.5 mM citric acid, 121 mM disodium hydrogen phosphate, 1 mM EDTA, 1 mM DTT and 0.01% CHAPS (v/v); pH5.8) and incubated overnight at 37°C. Intact proteins were removed from the sample following precipitation with 2 volume ACN (250 μl) as described previously [23]. Samples were dried down using vacuum centrifugation and re-suspended in 200 μl of loading buffer (20 mM citric acid, 0.1% (v/v) ACN and 0.1% v/v formic acid). Samples were further diluted 1:100 in loading buffer prior to analysis by SRM for peptide product hpvFAAN.

### Assay linearity

One picomole (pmole) of synthetic cleaved substrate (hpvFAAN) was serially diluted to obtain seven samples of varying concentrations ranging to 1 attomole (amole). These were spiked in 25 μl of plasma diluted fivefold in assay buffer. Samples were analysed by the SRM workflow described following ACN precipitation. 1 pmole to 1 amole of hpvFAAN peptide alone was also analysed by the SRM workflow.

### Measuring AEP activity in plasma using SRM-MS

25 μl of bone marrow or peripheral blood plasma from childhood ALL patients, peripheral blood plasma from healthy or ovarian cancer patients were diluted in assay buffer as described above. Samples were incubated with 4 mM of synthetic substrate peptide (hpvFAANDVSKvph) for 16 hr at 37°C. Following ACN precipitation samples were re-suspended in loading buffer as above and 10 μl of sample was used for analysis by LC-MS.

### Measuring AEP levels in plasma using ELISA

Total level of plasma AEP was measured using ELISA following manufacture's protocol; (Total Legumain DuoSet kit from R&D Systems). A standard curve for AEP activity was obtained by serial dilution of 2 ng/ml rAEP across the 96 well ELISA plate. Eight different concentrations of rAEP ranging from 0.015 to 2 ng/ml were used. Plasma samples were undiluted or diluted (1:10) in diluent reagent, supplied in the kit.

## SUPPLEMENTARY FIGURES AND TABLES



## References

[1] Anderson NL, Anderson NG (2002). The Human Plasma Proteome: History, Character, and Diagnostic Prospects. Molecular & Cellular Proteomics.

[2] Jacobs JM, Adkins JN, Qian W-J, Liu T, Shen Y, Camp DG, Smith RD (2005). Utilizing Human Blood Plasma for Proteomic Biomarker Discovery. Journal of Proteome Research.

[3] Grossklaus DJ, Smith JA, Shappell SB, Coffey CS, Chang SS, Cookson MS (2002). The free/total prostate-specific antigen ratio (%fPSA) is the best predictor of tumor involvement in the radical prostatectomy specimen among men with an elevated PSA. Urol Oncol.

[4] Whitehouse C, Solomon E (2003). Current status of the molecular characterization of the ovarian cancer antigen CA125 and implications for its use in clinical screening. Gynecol Oncol.

[5] Tirumalai RS, Chan KC, Prieto DA, Issaq HJ, Conrads TP, Veenstra TD (2003). Characterization of the low molecular weight human serum proteome. Mol Cell Proteomics.

[6] Dando PM, Fortunato M, Smith L, Knight CG, McKendrick JE, Barrett AJ (1999). Pig kidney legumain: an asparaginyl endopeptidase with restricted specificity. Biochemical Journal.

[7] Mathieu MA, Bogyo M, Caffrey CR, Choe Y, Lee J, Chapman H, Sajid M, Craik CS, McKerrow JH (2002). Substrate specificity of schistosome versus human legumain determined by P1-P3 peptide libraries. Mol Biochem Parasitol.

[8] Rawlings ND, Morton FR, Barrett AJ (2006). MEROPS: the peptidase database. Nucleic Acids Research.

[9] Patel N, Krishnan S, Offman MN, Krol M, Moss CX, Leighton C, van Delft FW, Holland M, Liu J, Alexander S, Dempsey C, Ariffin H, Essink M (2009). A dyad of lymphoblastic lysosomal cysteine proteases degrades the antileukemic drug L-asparaginase. J Clin Invest.

[10] Chen JM, Dando PM, Rawlings ND, Brown MA, Young NE, Stevens RA, Hewitt E, Watts C, Barrett AJ (1997). Cloning, isolation, and characterization of mammalian legumain, an asparaginyl endopeptidase. J Biol Chem.

[11] Haugen MH, Boye K, Nesland JM, Pettersen SJ, Egeland EV, Tamhane T, Brix K, Maelandsmo GM, Flatmark K (2015). High expression of the cysteine proteinase legumain in colorectal cancer – Implications for therapeutic targeting. European Journal of Cancer.

[12] Lin Y, Qiu Y, Xu C, Liu Q, Peng B, Kaufmann GF, Chen X, Lan B, Wei C, Lu D, Zhang Y, Guo Y, Lu Z, Jiang B, Edgington TS, Guo F (2014). Functional role of asparaginyl endopeptidase ubiquitination by TRAF6 in tumor invasion and metastasis. J Natl Cancer Inst.

[13] Ohno Y, Nakashima J, Izumi M, Ohori M, Hashimoto T, Tachibana M (2012). Association of legumain expression pattern with prostate cancer invasiveness and aggressiveness. World Journal of Urology.

[14] Strefford JC, van Delft FW, Robinson HM, Worley H, Yiannikouris O, Selzer R, Richmond T, Hann I, Bellotti T, Raghavan M, Young BD, Saha V, Harrison CJ (2006). Complex genomic alterations and gene expression in acute lymphoblastic leukemia with intrachromosomal amplification of chromosome 21. Proceedings of the National Academy of Sciences.

[15] Holland M, Castro FV, Alexander S, Smith D, Liu J, Walker M, Bitton D, Mulryan K, Ashton G, Blaylock M, Bagley S, Connolly Y, Bridgeman J (2011). RAC2, AEP, and ICAM1 expression are associated with CNS disease in a mouse model of pre-B childhood acute lymphoblastic leukemia. Blood.

[16] Wu M, Shao GR, Zhang FX, Wu WX, Xu P, Ruan ZM (2014). Legumain Protein as a Potential Predictive Biomarker for Asian Patients with Breast Carcinoma. Asian Pacific Journal of Cancer Prevention.

[17] Liu C, Sun C, Huang H, Janda K, Edgington T (2003). Overexpression of Legumain in Tumors Is Significant for Invasion/Metastasis and a Candidate Enzymatic Target for Prodrug Therapy. Cancer Research.

[18] Basurto-Islas G, Grundke-Iqbal I, Tung YC, Liu F, Iqbal K (2013). Activation of Asparaginyl Endopeptidase Leads to Tau Hyperphosphorylation in Alzheimer Disease. Journal of Biological Chemistry.

[19] Chen JM, Rawlings ND, Stevens RA, Barrett AJ (1998). Identification of the active site of legumain links it to caspases, clostripain and gingipains in a new clan of cysteine endopeptidases. FEBS Lett.

[20] Silva D, Cortez CM, Louro SR (2004). Quenching of the intrinsic fluorescence of bovine serum albumin by chlorpromazine and hemin. Braz J Med Biol Res.

[21] Kitteringham NR, Jenkins RE, Lane CS, Elliott VL, Park BK (2009). Multiple reaction monitoring for quantitative biomarker analysis in proteomics and metabolomics. J Chromatogr B Analyt Technol Biomed Life Sci.

[22] Stokvis E, Rosing H, Beijnen JH (2005). Liquid chromatography-mass spectrometry for the quantitative bioanalysis of anticancer drugs. Mass Spectrom Rev.

[23] Potier DN, Griffiths JR, Unwin RD, Walker MJ, Carrick E, Willamson AJ, Whetton AD (2012). An assessment of peptide enrichment methods employing mTRAQ quantification approaches. Anal Chem.

[24] Villanueva J, Nazarian A, Lawlor K, Yi SS, Robbins RJ, Tempst P (2008). A sequence-specific exopeptidase activity test (SSEAT) for “functional” biomarker discovery. Mol Cell Proteomics.

[25] Hortin GL, Sviridov D (2010). The dynamic range problem in the analysis of the plasma proteome. Journal of Proteomics.

[26] Gawenda J, Traub F, Lück HJ, Kreipe H, Wasielewski R (2006). Legumain expression as a prognostic factor in breast cancer patients. Breast Cancer Research and Treatment.

[27] Wu W, Luo Y, Sun C, Liu Y, Kuo P, Varga J, Xiang R, Reisfeld R, Janda KD, Edgington TS, Liu C (2006). Targeting Cell-Impermeable Prodrug Activation to Tumor Microenvironment Eradicates Multiple Drug-Resistant Neoplasms. Cancer Research.

[28] Chen JM, Fortunato M, Barrett AJ (2000). Activation of human prolegumain by cleavage at a C-terminal asparagine residue. Biochem J.

