# Supplementary material for: Development of a selected reaction monitoring mass spectrometry-based assay to detect asparaginyl endopeptidase activity in biological fluids
Source: Oncotarget. 2016 Sep 23;7(43):70822–31. doi: 10.18632/oncotarget.12224 (PMC5342591; doi:10.18632/oncotarget.12224)
Supplement: Supplementary file 1 [file oncotarget-07-70822-s001.pdf]

Development of a selected reaction monitoring mass spectrometry-based assay to detect asparaginyl endopeptidase activity in biological fluids

SUPPLEMENTARY FIGURES AND TABLES

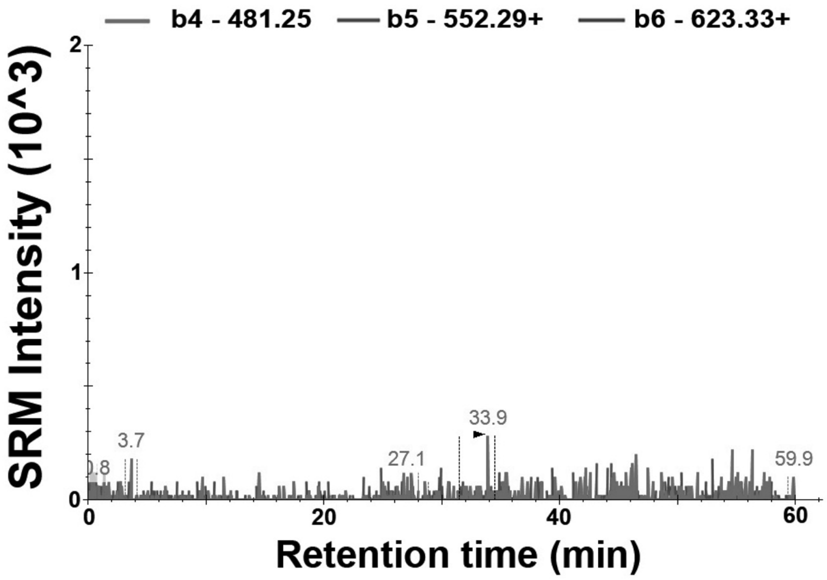

Supplementary Figure S1: SRM responses from putative endogenous peptides in plasma. Plasma samples from 10 healthy individuals were pooled and pH was adjusted to 5.8. Sample was incubated at 37°C for 16 hr without AEP synthetic substrate. Peptides were obtained from plasma after ACN precipitation. Sample was dried down and reconstituted with loading buffer. Sample was analyzed by SRM-MS in order to observe the background level of SRM signals from plasma.

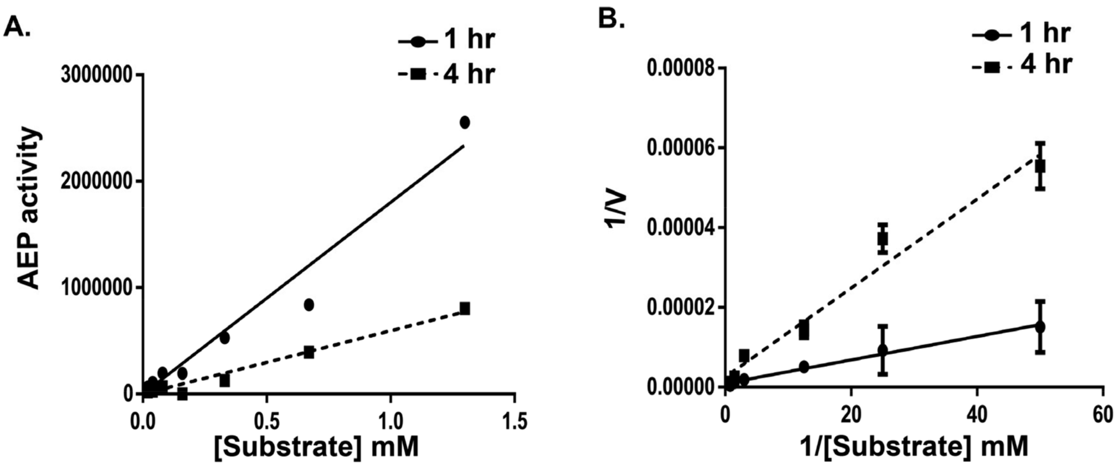

Supplementary Figure S2: Michaelis-Menten and Lineweaver-Burk plots showing AEP activity after 1 and 4 hr of incubation with the synthetic substrate. Rate of AEP activity was measured with different concentrations of substrate (hpvFAANDVSKvph) ranging from 0.02 mM to 1.3 mM. SRM Peak areas were used to obtain the rate of reaction for AEP activity as shown in Michaelis-Menten Curve **A.** Lineweaver-Burk plot was generated to calculate the Km value (Km=0.3mM at 1 hr and 0.33mM at 4 hr) for the synthetic substrate **B.** V represents the amount of product formed per hour. Error bars shown are SD (n = 3).

Supplementary Table S1: Amount of AEP detected in peripheral blood plasma using ELISA

PlasmaSample (undiluted)	OD	Blank OD	Actual OD	AEP Conc. (ng/ml)	STDEV
N 1	0.3740	0.109	0.2650	0.4123	0.0177
N 2	0.3327	0.109	0.2237	0.3305	0.0231
N 3	0.8393	0.109	0.7303	1.3334	0.0471
N 4	0.2950	0.109	0.1860	0.2559	0.0072
N 5	0.7547	0.109	0.6457	1.1658	0.0329
N 6	0.3807	0.109	0.2717	0.4255	0.0291
N 7	0.3643	0.109	0.2553	0.3932	0.0151
N 8	3.6283	0.109	3.5193	6.8540	0.0681
N 9	0.2697	0.109	0.1607	0.2058	0.0198
N 10	0.2383	0.109	0.1293	0.1438	0.0140
OC 1	0.5527	0.109	0.4437	0.7660	0.0207
OC 2	0.2050	0.109	0.0960	0.0778	0.0038
OC 3	1.5680	0.109	1.4590	2.7757	0.0553
OC 4	0.2890	0.109	0.1800	0.2441	0.0125
OC 5	0.5273	0.109	0.4183	0.7158	0.0199
OC 6	0.2050	0.109	0.0960	0.0778	0.0066
OC 7	0.6553	0.109	0.5463	0.9692	0.0021
OC 8	0.4620	0.109	0.3530	0.5865	0.0141
OC 9	0.2630	0.109	0.1540	0.1926	0.0070
OC 10	0.3777	0.109	0.2687	0.4196	0.0012

Commercially available ELISA was performed to detect level of total AEP in peripheral blood plasma collected from 10 healthy individuals (N) and those with ovarian cancer (OC). Plasma samples were undiluted. n=3.

Supplementary Table S2: Amount of AEP detected in bone marrow plasma from patients with acute lymphoblastic leukaemia using ELISA

PlasmaSample (1:10 diluted)	OD	Blank OD	Actual OD	AEP Conc. (ng/ml)	STDEV
ALL1	0.0996	0.054	0.0457	0.2091	0.0046
ALL 2	0.0903	0.054	0.0363	0.1333	0.0005
ALL 3	0.1603	0.054	0.1063	0.7015	0.0030
ALL 4	0.428	0.054	0.3740	2.8741	0.0141
ALL 5	0.486	0.054	0.4320	3.3449	0.0088
ALL 6	0.068	0.054	0.0140	-0.0478	0.0043
ALL 7	0.965	0.054	0.9110	7.2329	0.0138
ALL 8	0.224	0.054	0.1703	1.2210	0.0032
ALL 9	0.09	0.0500	0.0400	0.3984	0.0072
ALL 10	0.124	0.050	0.0743	0.6448	0.0015
ALL 11	0.092	0.050	0.0427	0.4175	0.0046
ALL 12	0.0753	0.0500	0.0253	0.2931	0.0015
ALL 13	0.2403	0.0500	0.1903	1.4776	0.1075
ALL 14	0.105	0.0500	0.0550	0.5061	0.002

ELISA was performed to detect level of total AEP in bone marrow plasma collected from 14 patients diagnosed with childhood ALL. Plasma samples were diluted 1:10 in diluting reagent. n=3.
